# Multispecies Fisheries in the Lower Amazon River and Its Relationship with the Regional and Global Climate Variability

**DOI:** 10.1371/journal.pone.0157050

**Published:** 2016-06-17

**Authors:** Walter Hugo Diaz Pinaya, Francisco Javier Lobon-Cervia, Pablo Pita, Ronald Buss de Souza, Juan Freire, Victoria Judith Isaac

**Affiliations:** 1 Laboratory of Fisheries Biology and Management of Aquatic Resources, Federal University of Pará, Belém, Pará, Brazil; 2 Laboratory of Meteorology and Satellite Oceanography, Southern Regional Center for Space Research, National Institute for Space Research, Santa Maria, Rio Grande do Sul, Brazil; 3 Department of Evolutionary Ecology, National Museum of Natural Sciences, Madrid, Spain; 4 Department of Applied Economics, Faculty of Economics and Business Administration, University of Santiago de Compostela, Santiago de Compostela, Galicia, Spain; 5 Grupo de Recursos Marinos y Pesquerías, Universidad de A Coruña, A Coruña, Galicia, Spain; Institut Maurice-Lamontagne, CANADA

## Abstract

This paper aims to describe the spatial-temporal variability in catch of the main fishery resources of the Amazon River and floodplain lakes of the Lower Amazon, as well as relating the Catch per Unit of Effort with anomalies of some of the Amazon River, atmosphere and Atlantic Ocean system variables, determining the influence of the environment on the Amazonian fishery resources. Finfish landings data from the towns and villages of the Lower Amazon for the fisheries of three sites (Óbidos, Santarém and Monte Alegre), were obtained for the period between January 1993 and December 2004. Analysis of variance, detrended correspondence analysis, redundancy analysis and multiple regression techniques were used for the statistical analysis of the distinct time series. Fisheries production in the Lower Amazon presents differences between the Amazon River and the floodplain lakes. Production in the Amazon River is approximately half of the one of the floodplain lakes. This variability occurs both along the Lower Amazon River region (longitudinal gradient) and laterally (latitudinal gradient) for every fishing ground studied here. The distinct environmental variables alone or in association act differently on the fishery stocks and the success of catches in each fishery group studied here. Important variables are the flooding events; the soil the sea surface temperatures; the humidity; the wind and the occurence of El Niño-Southern Oscillation events. Fishery productivity presents a large difference in quantity and distribution patterns between the river and floodplain lakes. This variability occurs in the region of the Lower Amazon as well as laterally for each fishery group studied, being dependent on the ecological characteristics and life strategies of each fish group considered here.

## Introduction

Fishing is a major activity in the Amazon River since the origins of the earliest native communities in the region [1]. In the Lower Amazon, this activity is different from other regions due to the large amount of species explored, their production and their different impacts on each of the human communities present in the region [2]. The fishery in this region is essentially artisanal and based on a diversity of fishing methods, with different degrees of technological development. Different fishing tactics are frequently applied, depending on the target species and the local environment [3], contributing to increase the uncertainties of our understanding of the fisheries in the Amazon.

The estimated number of fish species in the Amazon ranges from 1500–2000 to 8,000 species according to different authors [4–5]. However, the commercially exploited species varies within a narrow range of six to twelve species that correspond to about 80% of the total fish biomass landed [2]. The composition of the catch is related to the specific environment that predominates where the fishery is made as well to the nature and costumes of the regional communities. This is well exemplified by the predominance of scale fishes relative to catfish in the Central Amazon region which is echoed in the fish supply of the local markets [2].

Fish species exhibit adaptative tactics to cope with the seasonal changes in the hydrological cycle in the regions where they occur: either the floodplain lakes and/or in the main course of the Amazon River [2]. In order to understand the dynamics and composition of these fish species, it is critical to document their adaptative tactics and, therefore, new research is needed to better understand the biological cycles, feeding strategies, metabolism, individual growth and development and migration behaviour of the Amazonian fish. [6–14].

The distribution and ecology of fishery resources in the Amazon region are determined by the natural surroundings, availability of environments, meteorological characteristics and variability of the hydrological cycle. With a higher discharge, the Amazon River floods its banks and expand itself over the surrounding floodplains [2]. Thus, the flooding dynamics is expected to act over the fishery dynamics. Early works show that there is a strong relationship between the Amazonian hydrological cycle and the local fish catches throughout the year [15]. Floodplains and wetland forests are extremely valuable in ensuring the success of Amazonian commercial fisheries, which leads to the consequent need for their conservation [16–19].

To ensure a sustainable fishery and its long-term conservation, the concept of managing landscape units should be considered: we ought to understand the resources within the environment as a whole and their variability within the river-flooding plains system. Likewise, when considering the climate variability and changes, the ecological approach taken to understand the fisheries should consider the meso to macro spatial scales. This is paramount if we aim to strengthen our public conservation policies and improve the management of the fisheries resources in the Amazon River Basin region. These aspects indicate the importance of understanding the direct interactions between the living resources and their environment [2].

Some authors have studied the variability of the hydrological cycle and its relationship with the dynamics, recruitment and catch of commercial species in inland waters, in diverse places in the world [20–32], but not in the Amazon River Basin. To contribute to this lack of knowledge, this paper aims to study the spatial-temporal variability in catch of the main fisheries resources in the Lower Amazon, considering the different aquatic environments, i.e., the Amazon River and the floodplain lakes. This paper also aims to describe the relationship between fishery productivity using the variable CPUE (Catch per Unit Effort) and anomalies of some environmental variables of the river-atmosphere-ocean system, determining the influence of the environment on the the success of the local fisheries.

## Materials and Methods

### Study area

**Fig 1:**
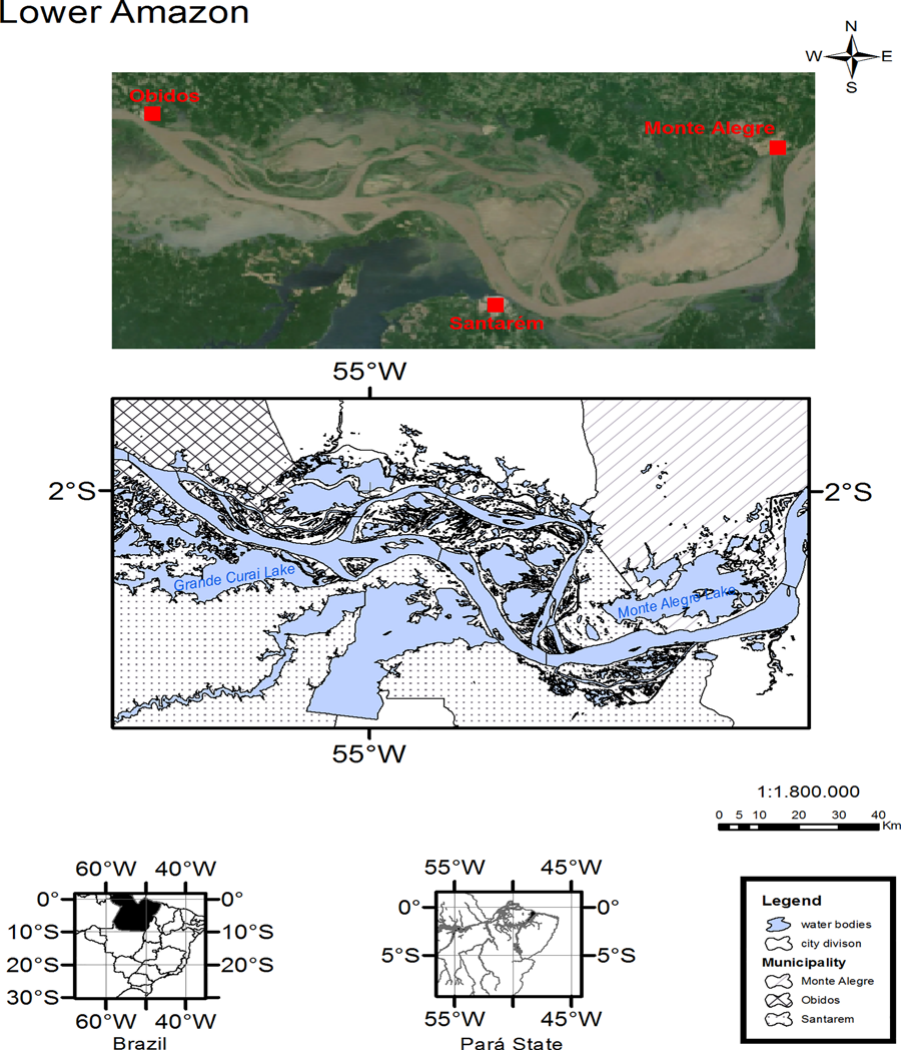
Study area. The Lower Amazon and the subdivision in three areas: Óbidos, Santarém and Monte Alegre.

The study area covers the fisheries grounds of Óbidos, Santarém and Monte Alegre, in the region of the Lower Amazon, between latitudes 1° 43’S– 2° 37’S and longitudes 55°55’W– 53°46’W (Fig 1).

### Materials

#### Fisheries data

Information on catch per species and fishing effort from January 1993 to December 2004 was collected on a daily basis for each fishing trip by means of interviews with the skippers or those responsible on the vessels that dock in the major catch landing ports of the study area [15]. The time series of fisheries data included 163,546 identification records of fishing units, where each record corresponds to a single fishing trip. With the selection of attributes–fishing boat and gill net–the series were reduced to 19,484 records for the river environment and 37,624 records for the floodplain lakes, only considering here the fisheries in the Lower Amazon. This selection is justified since it is known that gill net is the main fishing method in the Lower Amazon, and the fishing boats (boats with their own crew for catching fish) are the most representative units of this artisanal fleet, accounting for over 80% of all the fisheries production in the region [33]. A criteria for reducing the number of species studied here from all species caught in the Lower Amazon region included the use of a multivariate analysis (discussed later on this text). The analysis was performed over a number of fish families that represented about 80% of the local fish production in each of the two environments studied here: river and floodplain lakes.

Due to the low abundances and small importance on the regional fisheries, some fish families were not considered here. They are: Anostomidae, Serrasalmidae and Loricariidae at Óbidos (for the river environment); Loricariidae and Doradidae at Óbidos and Clupeidae at Monte Alegre (for the floodplain lakes). The most representative fish families of our study area were the Pimelodidae (in the Amazon Rover and especially during the dry season) and the Hypophthalmidae (in the lakes and especially during the wet season).

#### Hydrological data

The hydrological data of the Amazon River used here were the discharge (ARD) for Óbidos, water level (WL) for Santarém and a spatial average of rainfall (RF) for the Lower Amazon region were obtained from the Brazilian Water Management Agency (ANA, Agência Nacional de Águas– www.ana.gov.br). Time series at the monthly temporal resolution were obtained for the same period of the fishery data, covering January 1993 to December 2004. The time series were used for calculating the climatology means of discharge, water level and rainfall for the Lower Amazon River region during the period analysed here.

#### Meteorological and oceanographic data

Other meteorological data apart from rainfall were obtained from the NCEP/NCAR (National Centers for Environmental Prediction / National Center for Atmospheric Research) Reanalysis Project. We used data from both N/N Reanalysis [34] and Reanalysis 2 [35] databanks. Monthly averages were used, distributed in a Gaussian Grid with a spatial resolution of 1.8758 km x 1.9058 km (latitude/longitude). The variables studied here were zonal (u) and meridional (v) wind surface (10 m) anomalies, surface (2 m) specific humidity (SPFH), minimum air temperature (TMP), soil temperature (TMPsfc), potential evaporation (PEVPR), latent heat flux (LHF) and runoff (RUNOF). The meteorological data is publicly available on-line (http://www.esrl.noaa.gov/psd/data/gridded/).

Monthly sea surface temperature (SST) data were also obtained in a 4 × 4 km grid along the coast of Pará and Amapá states in the Brazilian Amazonia, as well as for the mouth of the Amazon River. This oceanographic variable was obtained from the “Optimum Interpolation Sea Surface Temperature Version 2—First Guess SST Field” dataset, processed with the NLSST (Non Linear Sea Surface Temperature) algorithm, available on the database of the Pathfinder project v.5. (PV5) developed by the NODC (National Oceanographic Data Center - https://podaac.jpl.nasa.gov/datasetlist?search=Pathfinder) and RSMAS (Rosenstiel School of Marine and Atmospheric Science,—University of Miami)

#### Climatological indices

In order to time correlate the oscillations found in the fisheries time series with climatological events possibly affecting the study region, we used time series of some known climatic indices. Owing to known linkages of climatic events such as the El Niño- Southern Oscillation (ENSO) and the variability of the SST fields in the Atlantic Ocean [36–40], we used the Multivariate ENSO Index (MEI), the North Atlantic Oscillation Index (NAO) and the Inter-Hemispheric SST Gradient of the Atlantic (GITA). The time series of MEI and NAO indexes were obtained from www.esrl.noaa.gov/psd/data/climateindices/list/. The GITA index was calculated by the diference between SST anomalies in the North and the South Atlantic Ocean [41].

### Methods

#### CPUE (Catch per Unit of Effort) estimation

In order to measure productivity, catch per unit of effort (CPUE) was estimated here for each fishery and species. CPUE is defined as the amount of fisheries resource caught by an effort unit employed. The CPUE was estimated by using catch in tonnes divided by the effort (number of fishermen x fishing days). The CPUE estimate was made by using the Jackknife method, described as [42]:

$$CPUE=\frac{\sum_{i=1}^{n}CiEi}{\sum_{i=1}^{n}E_{i}^{2}}$$ (1)

where *C*_*1*_ is the catch in a specific month and fishery, and *E*_*1*_ is the effort for the same month and fishery. The Jackknife method was selected since it is recommended for situations in which there is a lack of knowledge on the behaviour of the choosen variables, as is the case in the majority of fisheries studies [43].

The CPUE, on the other hand, will be considered as being related not only to catching the resource, but also to the availability of the fishery environment, i.e., both its catchability (Q) and its abundance (A), as shown below:

$$\begin{array}{l} \;CPUE\equiv f\left(A,Q\right)\;∴\\ CPUE\propto Q\;\to Q\equiv f\left(environment\right) \end{array}$$ (2)

Our Amazonian fisheries data were grouped according to the taxonomic family and the monthly value of the CPUE was estimated for each fishery site (Óbidos, Santarém and Monte Alegre) and fishery environment (river and floodplain lakes).

#### Market Index

There are two different fish markets in our study region. Industrial fish processing companies are located in Óbidos and Santarém while local consumption markets are mainly located in Santarém and Monte Alegre. This fact contributes for the fisheries dynamics in the Lower Amazon region. In order to improve our future multivariate statistical analysis, we took into account the fish market as well as the different fish sites and the environmental variables. A fish market index was produced here in order to account for the fisheries destination, aiming to take into consideration the economical drivers of the Lower Amazon fisheries. The market index is set to be 1 (one) when the monthly fish production was destined toward the industrial processing companies and 0 (zero) when it was destined toward the local consumption markets. The estimate of the market index was made using the following equations:

MKTRv=PIcatchtotalfishing (3)

MKTLk=HYcatchtotalfishing (4)

where, MKT_Rv_ is the marked index for the River Amazon environment; MKT_Lk_ is the marked index for the lake environment; PI is the catch of Pimelodidae and HY is the catch of Hypophthalmidae, the two more representative families in the two studied environments. Market indexes were computed for the two different environments and also for the different fishery locals.

#### Collinearity tests between the biological and envinronmental variables

In order to avoid possible distortions on our inferences as well as weak conclusions all our biological and environmental data were subject to a robust statistical method to test for the collinearity among all variables studied here. The method is described in [44]. When some of our variables are highly correlated we can opt on despise one or some of them aiming to not repeat our interpretations. Following [45], we used here the Pearson correlation method and used dispersion diagrams to analize the collinearity among our data.

**Fig 2:**
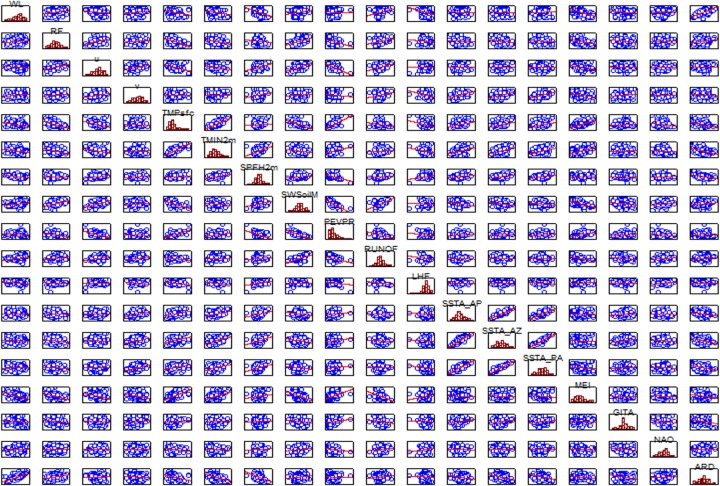
Dispersion diagram and correlations among the environmental variables. (ARD) Amazon River Discharge, (WL) Water Level, (RF) Rainfall, (u) Zonal Wind Component, (v) Meridional Wind Component, (TMPsfc) Surface Temperature, (TMIN2m) Air Temperature, (SPFH2m) Specific Humidity at 2 m height, (SWSoilM) Water Content of the Soil, (PEVPR) Potential Evaporation, (RUNOF) Runoff, (LHF) Latent Heat Flux, (SSTA) Sea Surface Temperature, (MEI) Multivariate ENSO Index, (GITA) Atlantic I*nter-Hemispheric Sea Surface Temperature Gradient*, (NAO) North Atlantic Oscillation, (AP) Continental Shelf of Amapá, (AZ) Continental Shelf of the Amazon, (PA) Continental Shelf of Pará.

The results from the Pearson correlation analysis are seen in Table 1 and Fig 2. As expected, the analysis indicates that the flooding pulse of the Amazon River floodplains is very correlated with the river's discharge (correlation coeficient, cc = 0.79). At the same time, the surface (soil) temperature is correlated to the air temperature (cc = 0.80) and with ENSO events (cc = 0.59). The SST anomalies of the continental shelf of Amapá, Amazon River mouth and Pará are highly correlated among them (cc > 0.90).

**Table 1:** Pearson’s correlation coeficients. Pearson’s correlation coeficients between environmental variables. (p < 0.05).

	ARD	WL	RF	u	v	TMPsfc	TMIN2M	SPFH2M	SWSoilM	PEVPR	RUNOF	LHF	SSTA_AP	SSTA_AZ	SSTA_PA	MEI	GITA	NAO
ARD	1.00																	
WL	0.63	1.00																
RF	0.24	0.20	1.00															
u	0.02	-0.12	0.36	1.00														
v	-0.11	0.16	0.12	0.27	1.00													
TMPsfc	-0.35	-0.26	-0.50	-0.40	-0.02	1.00												
TMIN2M	-0.44	-0.23	-0.34	-0.27	0.03	0.76	1.00											
SPFH2M	-0.45	-0.37	-0.12	0.31	0.21	0.07	0.43	1.00										
SWSoilM	0.17	0.21	0.41	0.49	0.09	-0.54	-0.18	0.25	1.00									
PEVPR	-0.04	-0.03	-0.39	-0.61	-0.12	0.69	0.30	-0.50	-0.76	1.00								
RUNOF	0.15	0.31	0.28	0.36	0.25	-0.23	0.06	0.14	0.63	-0.42	1.00							
LHF	-0.04	-0.29	-0.11	-0.01	-0.27	-0.28	-0.22	0.28	-0.05	-0.18	-0.22	1.00						
SSTA_AP	-0.25	-0.02	-0.05	0.00	0.39	0.32	0.35	0.29	-0.26	0.16	-0.08	-0.08	1.00					
SSTA_AZ	-0.35	-0.02	-0.15	-0.03	0.35	0.35	0.40	0.33	-0.24	0.18	-0.08	-0.06	0.89	1.00				
SSTA_PA	-0.34	0.05	-0.07	0.00	0.42	0.30	0.39	0.32	-0.16	0.12	0.00	-0.14	0.82	0.93	1.00			
MEI	-0.20	-0.45	-0.38	-0.34	-0.26	0.59	0.42	0.16	-0.49	0.45	-0.45	0.20	-0.05	-0.08	-0.12	1.00		
GITA	-0.28	-0.13	-0.20	-0.06	0.14	0.13	0.05	0.02	-0.19	0.16	-0.19	-0.03	0.20	0.24	0.12	-0.03	1.00	
NAO	0.06	0.10	0.03	0.01	-0.03	-0.19	-0.07	0.08	0.17	-0.16	0.14	0.18	-0.06	-0.01	-0.04	-0.06	-0.22	1.00

(ARD) Amazon River Discharge, (WL) Water Level, (RF) Rainfall, (u) Zonal Wind Component, (v) Meridional Wind Component, (TMPsfc) Surface Temperature, (TMIN2m) Air Temperature, (SPFH2m) Specific Humidity at 2 m height, (SWSoilM) Water Content of the Soil, (PEVPR) Potential Evaporation, (RUNOF) Runoff, (LHF) Latent Heat Flux, (SSTA) Sea Surface Temperature, (MEI) Multivariate ENSO Index, (GITA) Atlantic Inter-Hemispheric Sea Surface Temperature Gradient, (NAO) North Atlantic Oscillation, (AP) Continental Shelf of Amapá, (AZ) Continental Shelf of the Amazon, (PA) Continental Shelf of Pará.

After the collinearity tests, the environmental variables used here were reduced to the following: Discharge Anomaly of the Amazon River (ARD); Rainfall (RF); Water Level (WL); zonal (u) and meridional (v) components of the wind; Surface Temperature (TEMP); Air Humidity (SPFH); Runoff (RUNOF); Latent Heat Flux (LHF); sea Surface Temperature (SSTA); Climatic indexes MEI, GITA and NAO.

### Multivariate statistical analysis

Many distinct mathematical and statistical models have been applied into fisheries data. Among them, the multivariate regression analysis like the Autoregressive Integrated Moving Average (ARIMA) and the General Linear model (GLM) are highly recommended [46–53]. Some authors also employ efficiency models relating fish production with some environmental variables [54–56]. The Redundancy Analysis (RDA) is also a model largely employed in ecological studies to analyse data time series. This method when applyed to fisheries studies empirically assums that the several environmental variables regulate the fishery resources' dinamics as much as the fishery effort do [45]. This kind of analysis assumes a linear behavior for the variables studied allowing that the unkown variables fit into a linear regression model.

For all the variables analysed here, tests for normality were conducted and the data homocedasticity was analysed using the Shapiro-Wilk and Bartlett tests, respectively, accepted for the significant level of p < 0.05. The Analysis of Variance (ANOVA) test was applied aiming to test the variance in the monthly average CPUE according to the taxonomic family of the most abundant fish species in the Lower Amazon region. For the ANOVA, the factors established were the taxonomic families, the fisheries region, the months and the years of the fisheries.

Ordination was made for the three fishing grounds studies here (Óbidos, Santarém and Monte Alegre) as well as for their respective fishery environments (river and floodplain lakes) using the Detrended Correspondence Analysis (DCA) of the monthly averaged CPUE [57]. The CPUE values analysed here were logarithmically transformed before proceeding with the analysis [58]. DCA is a statistical tool mainly used to explore the data and show initial relationships in one source of data, although it can also be used to choose between linear or unimodal ordination methods [59]. This analysis made possible to graphically visualize the variation in the associations between our variables.

The relationships between the monthly averaged CPUE related to the taxonomic family and the environmental variables were analyzed using the RDA and the multiple regression. RDA was also employed using a matrix of explanatory variables (environmental variables) to quantify the variation in a matrix of response variables (CPUE), assuming linear relationships between all variables [60]. RDA is a tool that has been extensively used to analyze ecological relationships, e.g. between abundance data and environment variables. Environmental data are used to extract patterns from the explained variation only. RDA was he linear gradient analysis method. The focus scaling was on inter-species correlations and species score was divided by standard deviation. We also applied a fourth root transformation into our species data which were previously normalized and standardized. The forward selection of environment variables was made by manual selection with the best K of 24 variables and using Monte Carlo permutation tests with 9999 unrestrict permutations. The data set was then randomized and each variable was analyzed on its own.

The multiple regression method used here was the Backward Stepwise Method applied to a confidence level of 2 (F Fisher-Snedecor value). The dependent variables were the CPUE while the independent variables were the environmental variables and the market index. The fisheries data were transformed through the fourth root transformation. All regressions significant to p < 0.05 were considered here for future analysis. All the statistical analysis from the ANOVA and the multiple regression tests were made using the STATISTICA7.0^®^ software resulting in several statistical models which relate CPUE with the environmental variables and the market index. When performing the DCA we used the PCORD software run at the Natural Sciences Museum in Madrid, Spain. The RDA was run using the Canoco program for Windows 4.54 [60].

## Results

### Fishing in the Lower Amazon and specific composition

#### Fisheries production

For the period 1993–2004, the estimated total fisheries production in the Lower Amazon region was 17,482 t, with a monthly mean of 122 t (std = 49 t). August and September of 1994, 1995, 2001 and 2002 were the months with the highest catches. These months were part of interannual periods of ebb-drought in the Amazon River Basin. December 2002 and 2003, January and February 2000 and 2004 were the months with the lowest catches in the study region. These periods coincide with the wet period of the study region. The most caught species (81.4% of total) were *Hypophthalmus marginatus* and *H*. *edentatus* (26.9%), *Brachyplatystoma rousseauxii* (11.6%), *Prochilodus nigricans* (8.6%), *Plagioscion sp*. and *Pachypops sp*. (5.4%), *Pseudoplatystoma fasciatum* and *P*. *tigrinum* (5.3%), *Liposarcus pardalis* (5.0%), *Semaprochiloduns taeniurus* and *S*. *insignis* (4.5%), *Pimelodina flavipinnis* (3.8%), *Schizodon fasciatum* and *Leporinus trifasciatus* (3.4%) and *Brachyplatystoma vaillanti* (3.2%). Each fishing trip presented a mean fishing effort of 6 fishermen (std = 2 fishermen) and 4 fishing days (std = 1 day).

**Fig 3:**
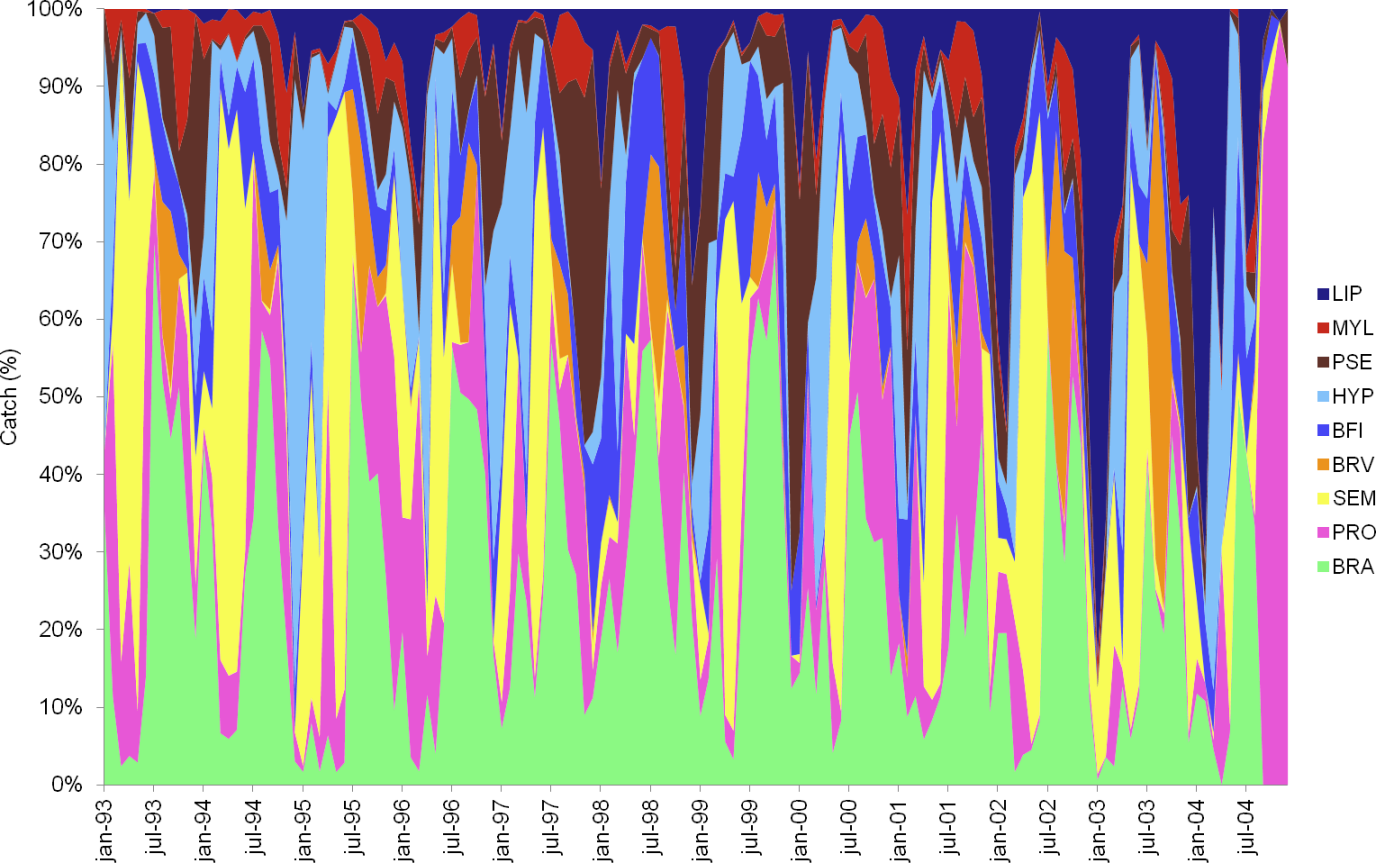
Monthly fishery production by species in the Amazon River. Monthly fishery production by species in the Amazon River near the fishery grounds of Óbidos, Santarém and Monte Alegre, from January 1993 to December 2004. (BRV) *Brachyplatystoma vaillanti*, (BRA) *Brachyplatystoma rousseauxii*, (SEM) *Semaprochiloduns taeniurus* and *S*. *insignis*, (BFI) *Brachyplatystoma filamentosum*, (HYP) *Hypophthalmus marginatus* and *H*. *edentatus*, (MYL) *Mylossoma duriventre*, *Myleus schomburgki* and *Metynnis argenteus*, (LIP) *Liposarcus pardalis*, (PRO) *Prochilodus nigricans*, (PSE) *Pseudoplatystoma fasciatum* and *P*. *tigrinum*.

As expected, our results show that the fisheries production in the lotic environment of the Lower Amazon River was cyclic in nature. The annual peak production happened in the period from August to October (drought-flood season), with less expressive values from December to February (wet season). The years 1995, 1999, 2001 and 2002 stood out for having higher catches, while the years 1997, 2000 and 2003 presented the lower catch values (Fig 3). Out of all the species caught in the lotic environment, the most expressive ones during the studied period were: *Brachyplatystoma rousseauxii* (28.0%), *Prochilodus nigricans* (11.1%), *Semaprochiloduns taeniurus* and *S*. *insignis* (8.7%), *Brachyplatystoma vaillanti* (7.6%), *Brachyplatystoma filamentosum* (7.0%), *Pseudoplatystoma fasciatum* and *P*. *tigrinum* (5.6%), *Hypophthalmus marginatus* and *H*. *edentatus* (5.5%), *Mylossoma duriventre*, *Myleus schomburgki* and *Metynnis argenteus* (4.2%) and *Liposarcus pardalis* (3.8%). These species accounted for 81.4% of the catches in the analyzed period, accounting for a total of 4,700 t with a monthly mean of 33 t (std = 28 t).

**Fig 4:**
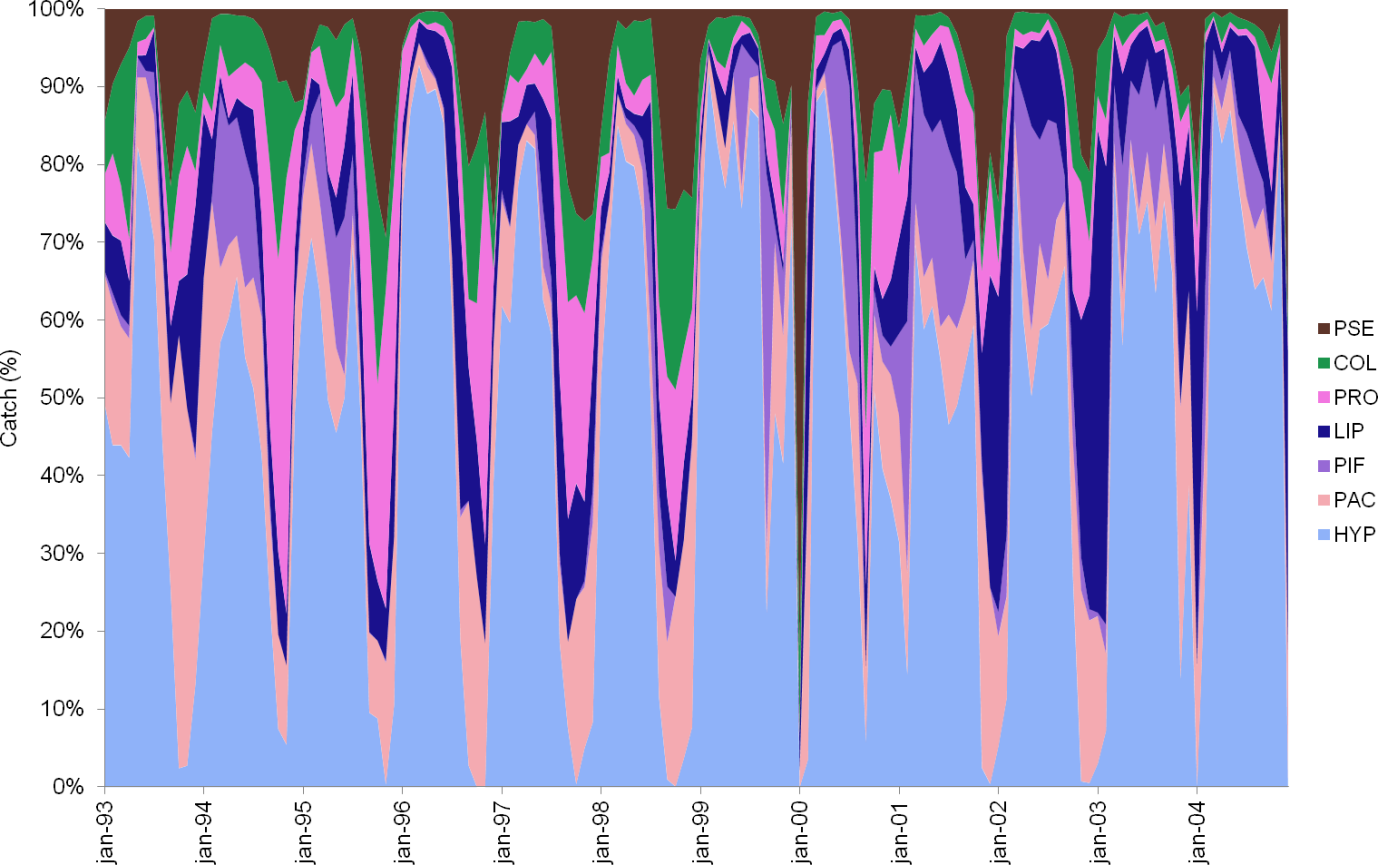
Monthly fishery production by species in floodplain lakes. Monthly fishery production by species in floodplain lakes near the fishery grounds of Óbidos, Santarém and Monte Alegre, from January 1993 to December 2004. (HYP) *Hypophthalmus marginatus* and *H*. *edentatus*, (LIP) *Liposarcus pardalis*, (PRO) *Prochilodus nigricans*, (PIF) *Pimelodina flavipinnis*, (PAC) *Plagioscion* spp. and *Pachypops* spp. (PSE), *Pseudoplatystoma fasciatum* and *P*. *tigrinum*, (COL) *Colossoma macropomum*.

In the lentic environment associated to the Amazon River, a rising trend was noted in total fish production, contrary to what commonly occurs in rivers. The years from 2001 to 2003 had the highest fish production, while the lowest productions were recorded in 1993 and from 1997 to 1999 (Fig 4). The wet period between February and March presented the highest values while the lowest values were related to the Amazonian drought-flood period, from October to December. The target species most caught in floodplain lakes during the study period were *Hypophthalmus marginatus* and *H*. *edentatus* (50.1%), *Plagioscion* spp. and *Pachypops* spp. (7.3%), *Pimelodina flavipinnis* (7.1%), *Liposarcus pardalis* (6.1%) and *Prochilodus nigricans* (4.7%), *Colossoma macropomum* (3.5%) and *Pseudoplatystoma fasciatum* and *P*. *tigrinum* (3.2%). These species accounted for 82.2% of the total production of 12,782 t in the study period. The fish production monthly mean was 89 t (std = 43 t).

**Fig 5:**
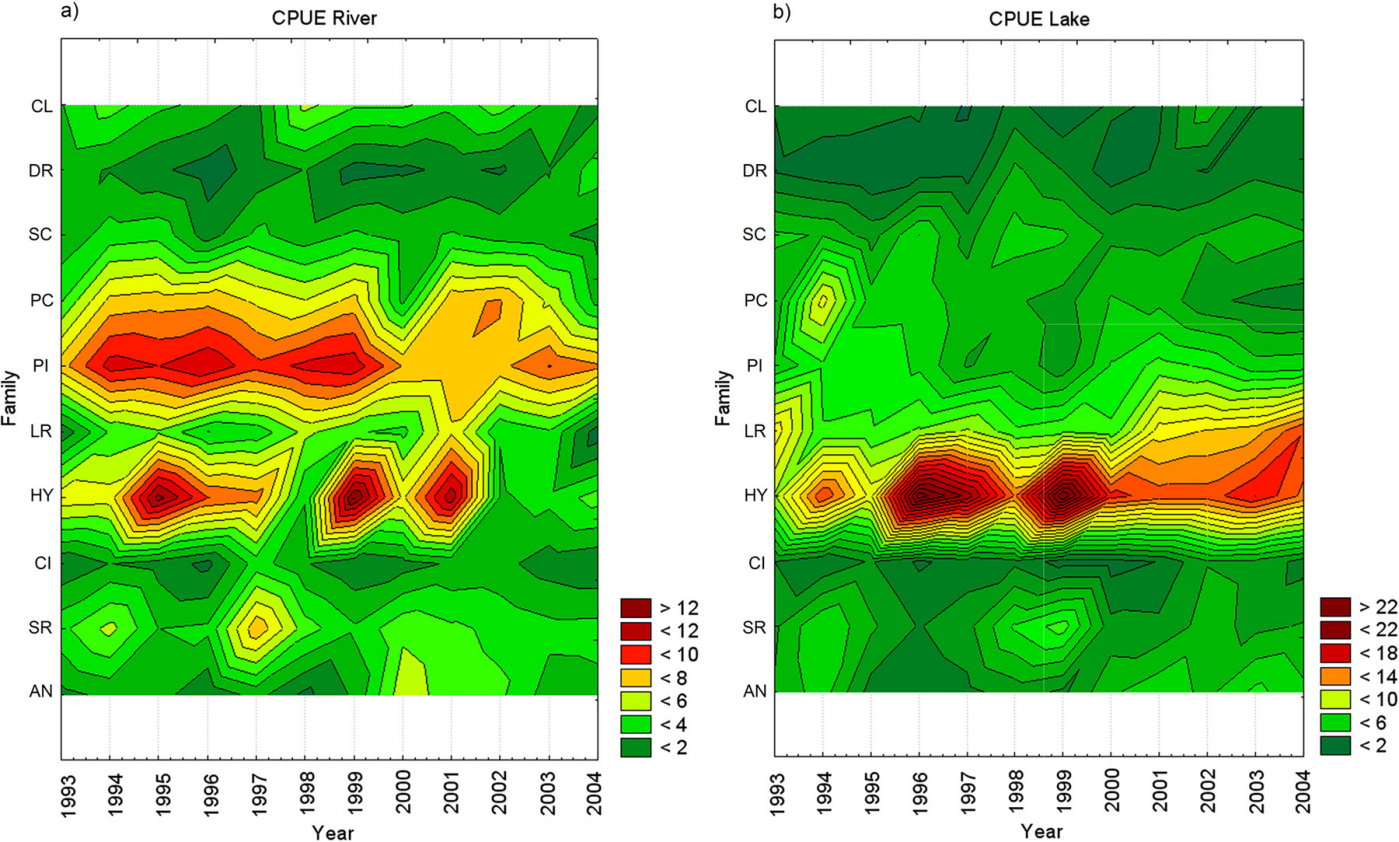
Temporal variation in the average CPUE by fish family. Diagram showing temporal variation in the average CPUE by taxonomic family in the catches in the (a) Amazon River, and in (b) floodplain lakes in the Lower Amazon, from January 1993 to December 2004. (HY) Hypophthalmidae, (PI) Pimelodidae, (SC) Sciaenidae, (PC) Prochilodontidae, (LR) Loricariidae, (SR) Serrasalmidae, (AN) Anostomidae, (CI) Cichlidae, (CL) Clupeidae, (DR) Doradidae.

When we group the species caught into their taxonomic families, the presence of 20 families of five orders are noted. The most important families are the Hypophthalmidae, Pimelodidae, Sciaenidae, Prochilodontidae, Loricariidae, Anostomidae, Cichlidae, Clupeidae and Doradidae. The evolution of the CPUE by taxonomic family caught in the Lower Amazon indicates an interannual variation and specificities that vary according to the fishery environment. The families Pimelodidae are mostly caught in the river (Fig 5A) while Hypophthalmidae are mostly caught in floodplain lakes (Fig 5B). These two taxonomic groups account for a higher CPUE observed in 1996 and 1999.

During the period of this study, the catch and CPUE varied according to the environment and the fishing area not always being directly proportional (Table 2). To illustrate this, in the river environment of Santarém, the most caught family was Pimelodidae with a monthly average of 12.16 t, while the family with the highest CPUE was Hypophthalmidae, with, 10.44 kg·fisherman^-1^·day^-1^ and a catch mean of only 787 kg per month. In the case of floodplain lakes, the highest catch was of Hypophthalmidae in Óbidos (18.15 t per month) which coincides with the highest CPUE (16.40 kg·fisherman^-1^·day^-1^). CPUE for the same Hypophthalmidae family was also very high in Monte Alegre and Santarém (16.33 kg·fisherman^-1^·day^-1^ and 15.10 kg·fisherman^-1^·day^-1^ respectively). In the lake environment, the Loricaridae family presented a high CPUE (10.83 kg·fisherman^-1^·day^-1^), but ranks in low position among the most caught families in lakes (2.06 t per month). We conclude that the fishing effort was not always directly related to the total catch.

**Table 2:** Fish production and CPUE per taxonomic family. Monthly averaged fish production (kg) and CPUE (kg·fisherman^-1^·day^-1^) per taxonomic family and fish caught and by artisanal driftnet fishery environment in the Lower Amazon.

	Obidos				Santarem				Monte Alegre			
	River		Lake		River		Lake		River		Lake	
	Catch	CPUE	Catch	CPUE	Catch	CPUE	Catch	CPUE	Catch	CPUE	Catch	CPUE
PI	4,832	9.30	2,531	4.90	12,161	9.37	5,533	5.05	3,719	8.87	5,599	6.94
PC	462	6.27	433	4.49	2,093	7.41	1,949	4.70	355	4.78	1,846	4.84
SR	307	3.61	592	4.47	1,676	5.18	2,185	4.01	309	3.74	1,695	4.78
HY	466	4.17	18,149	16.40	786	10.44	12,745	15.10	1,096	7.53	10,000	16.33
CL	108	1.00	79	1.00	495	1.00	359	1.00	74	1.00	631	1.00
DR	116	1.00	155	1.00	277	1.00	403	1.00	215	1.00	354	1.00
AN	77	1.05	653	3.29	432	4.49	2,909	5.63	54	2.53	717	3.64
SC	86	1.91	1,386	4.28	319	3.71	4,546	4.82	105	2.31	2,242	4.47
CI	23	0.86	193	2.79	205	2.18	996	2.64	91	2.16	631	2.21
LR	3	0.23	202	4.83	125	6.04	2,063	10.83	100	4.63	4,309	13.44

(PI) Pimelodidae, (PC) Prochilodontidae, (SR) Serrasalmidae, (HY) Hypophthalmidae, (CL) Clupeidae, (DR) Doradidae, (AN) Anostomidae, (SC) Sciaenidae, (CI) Cichlidae, (LR) Loricariidae.

#### Monthly and annual fishing pattern

**Fig 6:**
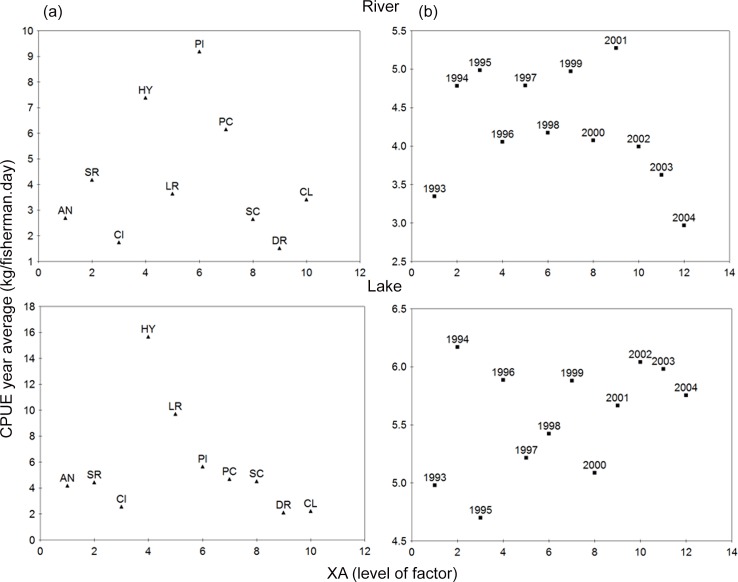
ANOVA for CPUE by target family and year. Graphic illustration of the results of the ANOVA test for the average CPUE for fisheries in the river (above) and floodplain lakes (below) in the Lower Amazon, by taxonomic family and year. (XA) level of the factor, (a) family and (b) year.

The ANOVA results for the correlations between the monthly averaged CPUE and four spatial-temporal variables (year, month, fishing ground and taxonomic family, Table 3) show that all the variables considered were found significant (p < 0.5) except for the correlation between month and fishing ground in floodplain lakes (Table 3, correlation Lake 2*3). Fig 6 presents a visual illustration of the ANOVA results for the CPUE correlations with the taxonomic family and the year for both the river and lake environments. Fig 6A indicates that in the river, the family Pimelodidae presents the highest CPUE followed by the Hypophthalmidae and Prochilodontidae families. In the floodplain lakes, Hypophthalmidae's CPUE stands out followed by Loricariidae's. The interannual variability can be accessed through the ANOVA graphics seen in Fig 6B where the year of 2001 (2004) is the one when the maximum (minimum) CPUE was produced for the river environment. In the floodplain lakes, maximum (minimum) CPUE was produced in 1994 (1995). The big discrepancies in CPUE between one year to the other will be discussed later on this text.

**Table 3:** ANOVA statistics between CPUE and the spatial-temporal variables. ANOVA results between monthly averaged CPUE and spatial-temporal variables.

	River		Lake	
	F	p	F	p
{1}Y	2.95	0.00	2.12	0.02
{2}M	8.06	0.00	3.08	0.00
{3}REG	38.53	0.00	15.34	0.00
{4}FM	43.82	0.00	196.60	0.00
1*2	1.25	0.04	1.45	0.00
1*3	2.19	0.00	3.62	0.00
1*4	1.49	0.00	3.76	0.00
2*3	2.23	0.00	0.88	0.62
2*4	2.75	0.00	3.28	0.00
3*4	4.44	0.00	8.82	0.00

The denotation n*m means the correlation between variable n and m. (Y) year, (M) month, (REG) fishing ground, (FM) taxonomic family.

#### Management of fishing patterns

**Fig 7:**
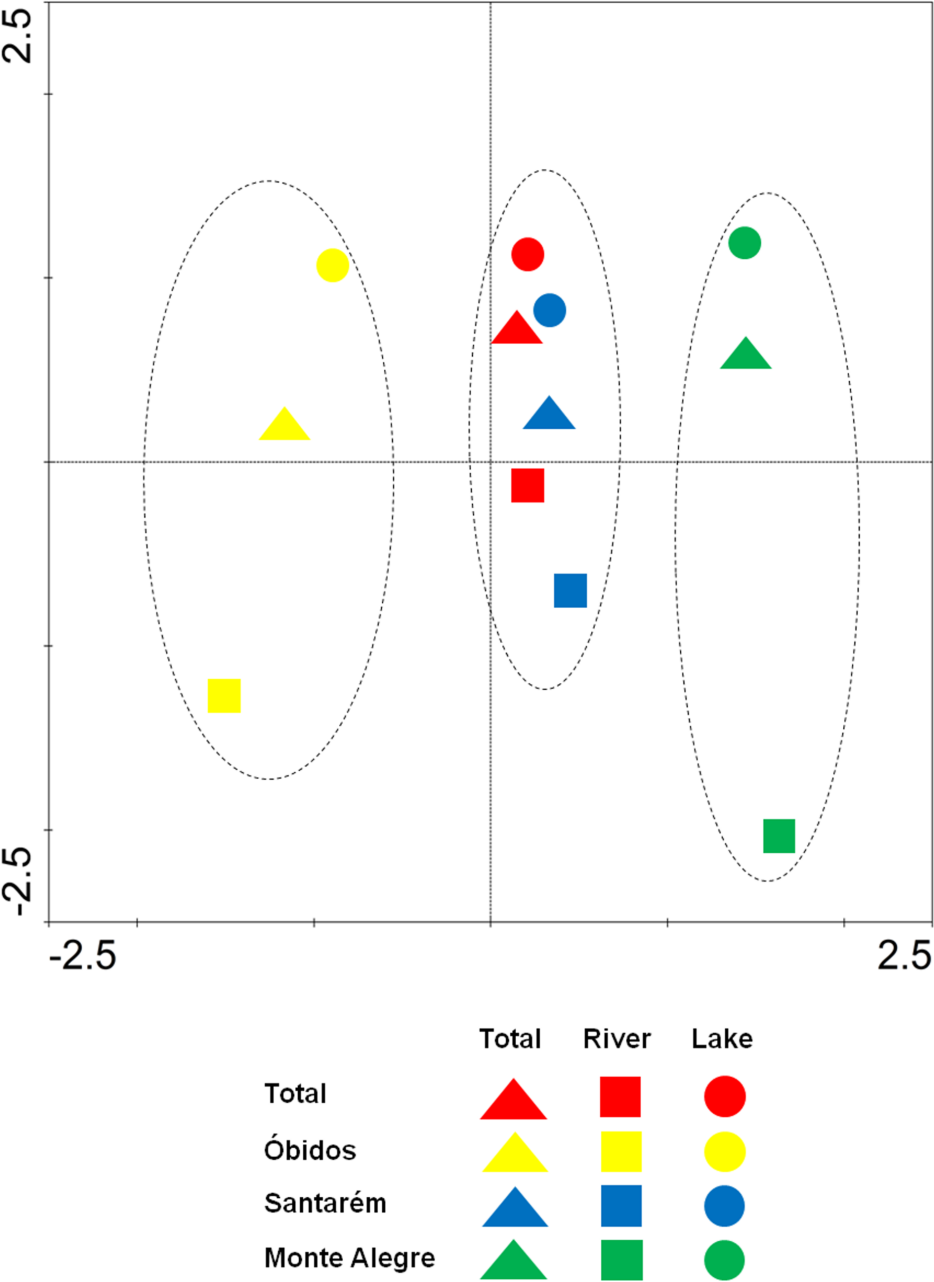
DCA for CPUE by fishery ground and environment. Graphic illustration of the DCA analysis for the monthly averaged CPUE in the Lower Amazon grouped by its fisheries grounds and the river or floodplain lakes environments.

The DCA analysis indicated that there was a difference between fishing patterns in the Lower Amazon in respect to the fishery activity and the the river or floodplain lakes environments. In Fig 7 it is possible to observe a clear separation in fishery activity (in the horizontal axis) according to the fishery grounds where the activity occurs (in the vertical axis), with Óbidos and Monte Alegre located on the extremes of the graph and Santarém located in an intermediate position between the other two. A separation is also seen between the fisheries carried out in the river and in floodplain lakes which are located in both extremes of the vertical axis (Fig 7).

**Fig 8:**
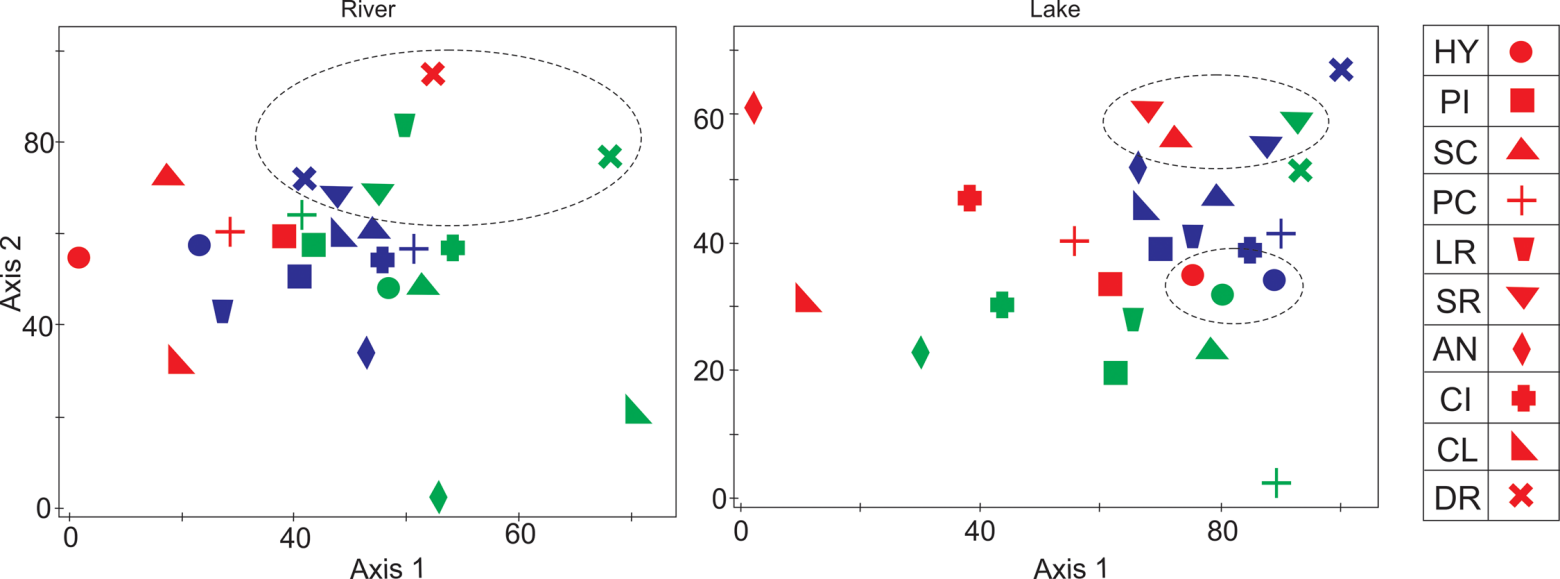
DCA analysis of the CPUE by target families. Graphic illustration of the first and second main component of the DCA analysis of the monthly average CPUE by target families caught in the Lower Amazon by fishery and environment. (HY) Hypophthalmidae, (PI) Pimelodidae, (SC) Sciaenidae, (PC) Prochilodontidae, (LR) Loricariidae, (SR) Serrasalmidae, (AN) Anostomidae, (CI) Cichlidae, (CL) Clupeidae, (DR) Doradidae; fishery environment in river (empty) and floodplain lake (full); the fishery of Óbidos (red), Santarém (blue), Monte Alegre (green). Axes 1 and 2 are the first and second main component of the data.

Apart from the existing legal management among the distinct fisheries of distinct species, the first and second main components of the DCA analysis showed that there are similar statistical patterns for some fishing systems, such as the catch of the Pimelodidae and Doradidae families in the Amazon River and the catch of the Hypophthalmus and Serrasalmidae families in the floodplain lakes (Fig 8).

### The relationship between fisheries production and climatic variability

**Fig 9:**
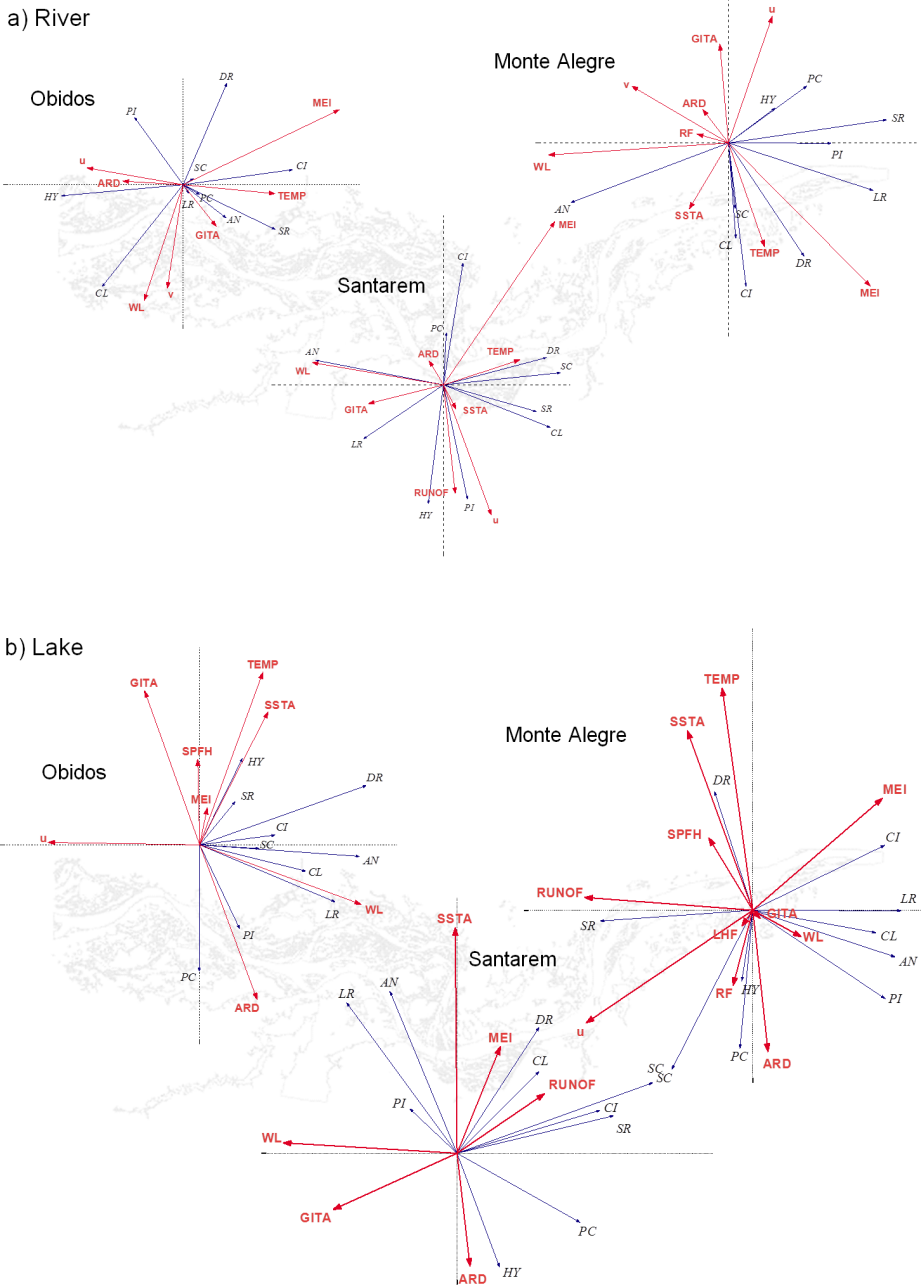
RDA of the CPUE in relation to the environmental variables. Graphic illustration of the RDA analysis of the monthly average CPUE in the river (a) and floodplain lakes (b) of the Lower Amazon region for all taxonomic families with respect to the environmental variables. Black arrows are related to the taxonomic families while the red arrows represent the several environmental variables considered here. (HY) Hypophthalmidae, (PI) Pimelodidae, (SC) Sciaenidae, (PC) Prochilodontidae, (LR) Loricariidae, (SR) Serrasalmidae, (AN) Anostomidae, (CI) Cichlidae, (CL) Clupeidae, (DR) Doradidae, (ARD) the Amazon River discharge, (WL) the Amazon River level, (u, v) zonal and meridional components of the wind, (TEMP) surface temperature, (SPFH) specific humidity of the air, (RUNOF) surface runoff, (LHF) latent heat flux, (GITA) Inter-Hemispheric SST Gradient of the Atlantic, (NAO) North Atlantic Oscillation Index, (MEI) Multivariate Climatic Index of ENSO events.

Fig 9 presents a graphic illustration of the RDA analysis used here to model the relationships between the fisheries production described by the CPUE by taxonomic family and the climatic variability in the Lower Amazon region described by the environmental variables. The analysis was also separated into the river (Fig 9A) and floodplain lakes (Fig 9B). Differences between the number of variables presented in each of the 6 graphics of Fig 9 are due to the fact that only statistically significant correlations are presented.

A general interpretation of Fig 9 should be made considering the angles between the black and arrows which represent respectively the taxonomic families and the environmental variables: angles close to 0° or 180° represent correlations close to 1 or -1, respectively. The arrows' lengths are also proportional to the intensity of the correlations.

We can see in Fig 9A, for example, that the monthly CPUE of the family Hypophthalmidae (HY) in the river environment of Óbidos was positively correlated to the Amazon River discharge (ARD) and negatively correlated to the surface temperature (TEMP). The contrary of that was found for the taxonomic family Serrasalmidae (SR): CPUE was positively correlated to the TEMP and negatively correlated to ARD. The same kind of analysis can be made for Santarém and Monte Alegre river environments. In Santarém there is a clear and strong inverse correlation between the CPUE of HY and the ENOS index MEI and a good direct correlation between the CPUE of HY and the sea surface temperature (SSTA). The productivity of HY in Monte Alegre was also inversely correlated to the SSTA. In this last locality we noticed a clear, strong inverse correlation between the CPUE of the Cichlidae family (CI) and the Inter-Hemispheric SST Gradient of the Atlantic (GITA).

Fig 9B presents the RDA analysis made between the CPUE of all taxonomic families and the environmental variables for the floodplain lakes. In Óbidos, for example, a noticeable inverse correlation occurs between the CPUE of both Loricariidae (LR) and Anostomidae (AN) families with the zonal component of the wind (u). Still in Óbidos, Prochilodontidae (PC)'s CPUE is inversely correlated to TEMP. The same occurs between Pimelodidae (PI)'s CPUE and GITA. In Santarém and Monte Alegre HY inversely correlates with MEI and SSTA.

Another way to analyze the relationship between CPUE and the environmental variables is to use multiple regression models. Results of this analysis seen in Table 4 show that the variable market (related to the final destination of the fisheries) is the most important one for the river environment catches in comparison to the lakes and especially in Óbidos and Santarém. The best adjusted model for this analysis was at the river environment between CPUE of Pimelodidae in Santarém with 64.81% of certainty (p = 0.0000) and in Monte Alegre with 59,20% of certainty (p = 0.0000). Considering the lake fisheries, the higher determination coefficients were found for the Loricaridae (LR, R^2^ = 0.2713, p = 0.0000), Cichlidae (CI, R^2^ = 0.2106, p = 0.0000) and Doradidae (DR, R^2^ = 17.77, p = 0.0001) families, respectively in Óbidos, Santarém and Monte Alegre.

**Table 4:** Multiple regression model. Best significant results for the CPUE of each taxnomic family, fishing ground and fishing environment from multiple regression. Significant values p < 0.05 and 2 degrees of freedom.

**RIVER**						
		R^2^	*p*	Ɛ std of Estimate	Interception	Model Equation
OBIDOS	SR	0.2669	0.00000	2.11580	2.0878	SR = 2.0878 + 0.275*MEI—0.41*MKT
	HY	0.2654	0.00000	2.50530	2.4609	HY = 2.4609 + 0.361*ARD + 0.195*SSTA + 0.287*RUNOF—0.38*MKT
	PI	0.2348	0.00001	1.97816	2.9799	PI = 2.9799–0.25*WL + 0.207*RF—0.20*v + 0.261*MKT
	PC	0.1807	0.00144	4.92976	4.5017	PC = 4.5017–0.22*MKT
	SC	0.2307	0.00016	1.40274	1.1752	SC = 1.1752–0.26*ARD + 0.284*WL + 0.369*u + 0.256*MEI—0.33*MKT
	DR	0.1092	0.01761	0.51768	0.3036	DR = 0.3036–0.26*TEMP + 0.306*MEI -0.20*MKT
	CL	0.2493	0.00001	1.22029	1.1025	CL = 1.1025 + 0.169*SSTA—0.36*MEI—0.27*RUNOF—0.25*MKT
SANTAREM	AN	0.1313	0.00147	1.12772	1.1578	AN = 1.1578–0.23*TEMP—0.20SPFH + 0.267*SSTA
	HY	0.1291	0.00067	5.13092	6.1742	HY = 6.1742–0.29*MKT
	LR	0.1605	0.00047	2.03290	2.6633	LR = 2.6633 + 0.265*TEMP + 0.190*SPFH—0.37*MEI—0.25*MKT
	PI	0.6481	0.00000	1.07017	0.3926	PI = 0.3926 + 0.161*ARD—0.26*WL—0.18*RF -0.13*SPFH + 0.223*SSTA—0.25*MEI + 0.706*MKT
	PC	0.0897	0.04213	2.43577	2.8433	PC = 2.8433–0.21*MKT
	SC	0.1147	0.01825	0.67738	1.1215	SC = 1.1215–0.28*WL—0.19*MKT
MONTE ALEGRE	AN	0.2895	0.00000	1.21969	0.2493	AN = 0.2493–0.23*u—0.43*LHF
	SR	0.1018	0.10478	1.15839	1.2407	SR = 1.2407–0.29*WL—0.19*LHF
	CI	0.2027	0.00002	0.60253	0.6249	CI = 0.6249–0.37*ARD + 0.233*WL—0.21*MKT
	HY	0.1961	0.00003	3.17750	3.5757	HY = 3.5757–0.21*SSTA + 0.210*GITA—0.27*MKT
	LR	0.1383	0.00090	1.95819	1.9148	LR = 1.9148 + 0.214*MEI—0.22*MKT
	PI	0.5920	0.00000	1.19065	0.8520	PI = 0.8520–0.29*WL—0.22*u—0.21*TEMP—0.26*LHF—0.15*RUNOF + 0.753*MKT
	PC	0.0935	0.03399	1.68062	1.5994	PC = 1.5995–0.29*WL—0.23*TEMP
	SC	0.1313	0.00332	0.86496	0.8101	SC = 0.8101–0.25*TEMP + 0.355*SPFH—0.25*LHF
	DR	0.1408	0.00731	1.59834	0.4593	DR = 0.4593 + 0.259*MEI
	CL	0.0738	0.02968	1.02307	0.2611	CL = 0.2611–0.18*LHF
		R^2^	*p*	Ɛ std of Estimate	Interception	Model Equation
OBIDOS	AN	0.1801	0.0002	1.1836	0.5663	AN = 0.5663–0.41*u—0.27*TEMP + 0.267*SSTA
	SR	0.1402	0.1058	1.5357	1.7031	SR = 1.7031–0.27*WL + 0.202*RF—0.23*u—0.28*MEI
	CI	0.0963	0.0202	1.3893	0.5563	CI = 0.5563 + 0.293*WL + 0.196*LHF
	HY	0.1787	0.0002	3.4437	3.5962	HY = 3.5962 + 0.256*ARD + 0.215*RF + 0.414*TEMP
	LR	0.2713	0.0000	1.7490	0.2788	LR = 0.2788–0.55*ARD + 0.518*WL—0.29*TEMP -0.32*SPFH + 0.310*MEI—0.25*GITA
	PC	0.1253	0.0133	2.5474	1.0287	PC = 1.0287 + 0.271*ARD—0.33*WL—0.29*u—0.42*TEMP
	SC	0.1467	0.0037	0.9441	1.3580	SC = 1.3580 + 0.277*ARD + 0.380*TEMP + 0.228*LHF
	DR	0.1764	0.0046	0.8924	0.4870	DR = 0.4870–0.31*ARD + 0.365*WL—0.21*u
SANTAREM	AN	0.1678	0.0003	0.7664	1.1736	AN = 1.1736–0.23*v + 0.237*SSTA—0.20*GITA + 0.197*MKT
	SR	0.1573	0.0027	0.5457	1.0539	SR = 1.0539 + 0.244*ARD—0.28*WL + 0.234*TEMP + 0.286*RUNOF
	CI	0.2106	0.0000	0.3291	0.7476	CI = 0.7476–0.35*WL—0.19*v
	HY	0.1457	0.0027	2.5188	2.8879	HY = 2.8879–0.26*WL + 0.188*MKT
	PI	0.0824	0.0171	0.7994	1.6174	PI = 1.6174–0.22*ARD—0.17*v—0.23*MEI
	SC	0.1891	0.0003	0.5361	1.1842	SC = 1.1842–0.19*v—0.24*GITA
	DR	0.1639	0.0018	0.6842	0.3724	DR = 0.3724 + 0.210*TEMP
MONTE ALEGRE	AN	0.1546	0.0069	0.9814	0.8905	AN = 0.8905 + 0.192*ARD—0.24*u
	SR	0.1317	0.0125	1.3418	1.2193	SR = 1.2193 + 0.209*RUNOF
	HY	0.0936	0.0081	3.8455	3.7127	HY = 3.7127 + 0.272*ARD + 0.170*LHF
	LR	0.1436	0.0006	1.5032	2.7674	LR = 2.7673 + 0.268*MEI + 0.178*GITA
	PI	0.1280	0.0155	1.4128	1.2851	PI = 1.2851–0.41*TEMP—0.22*SPFH—0.22*LHF + 0.234*SSTA + 0.281*MEI—0.18*GITA
	DR	0.1777	0.0001	0.7176	0.5794	DR = 0.5794–0.22*WL + 0.331*SPFH—0.22*LHF

(AN) Anostomidae, (CI) Cichlidae, (CL) Clupeidae, (DR) Doradidae, (HY) Hypophthalmidae, (LR) Loricariidae, (PC) Prochilodontidae, (PI) Pimelodidae, (SC) Sciaenidae, (SR) Serrasalmidae, (ARD) the Amazon River discharge, (WL) the Amazon River level, (*u*, *v*) zonal and meridional components of the wind, (TEMP) surface temperature, (SPFH) specific humidity of the air, (RUNOF) surface runoff, (LHF) latent heat flux, (GITA) Inter-Hemispheric SST Gradient of the Atlantic, (NAO) North Atlantic Oscillation Index, (MEI) Multivariate Climatic Index of ENSO events, (MKT) market index.

(R^2^) regression coefficient, (*p*) confidence level, (*Ɛ*) standard error of the estimative.

## Discussion

The ichthyofauna in the Amazon River is extremely heterogeneous in terms of life forms, behaviors and adaptations and depend on the size of the aquatic ecosystem available [61]. This is echoed by the fish catchability. Over 90% of the total catch of fish comes from the floodplains [62]. In different years, different groups of organisms may be more successful than others, considering their windows of opportunity [63] and phenological windows of susceptibility [64]. As a result, catch in the following seasonal periods can be higher for one target family in respect to another.

Although the river level may be an important variable to assess catch productivity forecasts [45], there are other environmental and socio-economic factors that may be equally important. The period and the intensity to which the floodplains stay wet have an influence on the fishery activity. For instance, we easily imagine that during the wet season the river's water level rising opens up a door to bring in more food, reproduction and growth areas as well as hiding areas in secure places that increases the fish success in lakes. The opposite happens during the dry season. When the door is open, the ichthyofauna is ready to populate the floodplains that were previously inundated. However, when the fish passes through the small canals leading to the lakes and when they occupy the hidden areas, the ecological pressure is at its maximum and only the most adapted species survive. When the door is closing it is time to abandon the floodplains and the individuals that cannot make it due to whatever factor are going to perish. When analyzing the fish productivity in the Amazon River with respect to the river's water level, [64] had noticed that after a perturbation in the system, the fish stock are no longer able to return to a stationary distribution.

In order to know what drives the fisheries variability we ought to know not only how the environment affects the fish biomass and the aquatic ecosystem as a whole, but also how all those factors interact one to each other [65]. The natural cycle of the flooding that drives the variability of the flooding area is easily observed in several tropical aquatic ecosystems like the Lower Amazon [57–58]. Each taxonomic family will behave in a distinct way towards the environmental variability adapting its survival rate and reproduction success. Human forcing as well as the environment will drive the fishing boats accessibility, the catchability and the fish production itself [59].

We might expect that the effects of the flooding pulse should be stronger during the wet season, but [53] observed that the effects of the dry season on the multispecies fisheries in the Amazon are equally important. The authors indicated that the fisheries dynamics in the river and floodplains areas of the Amazon is not simply dependent on the inundation pulse.

In the Amazon at the start of the flooding, the level of the river is practically stable. The aquatic environments on the floodplain predominate where a substantial part of the ichthyofauna is dispersed in search of food [10, 19, 33–34, 66–70]. During this period, fishing is more difficult and the catchability of target species is reduced because of the considerable dispersion of the individuals, arising from the increase of habitats and hidden areas. During ebb, fish begin to group together due to the contraction of the aquatic environment [2, 9]. In this period, catching is intense in the floodplain lakes and rivers. Both the floodplains and the forest are used by species of the families Pimelodidae and Sciaenidae since such fishes predate on other species that are leaving these areas during this period [7]. During the dry season, fish are forced to move to areas where water is abundant in the river channels [71]. However, not all Amazonian fishing boats are able to operate in this environment. During this period, there is a concentration of Pimelodidae catches in the river. At the end of the dry season, fisheries production of Serrasalmidae (genus *Colossoma*) and Prochilodontidae (genus *Prochilodus*) are intensified. At wet season, catch is concentrated into target families of lake-living species that have a life cycle mainly associated to the floodplain lakes or lake systems [72] such as Pimelodidae (genuses *Pimelodina* and *Pseudoplatystoma*), Sciaenidae (genus *Plagioscion*), Loricariidae (genus *Liposarcus*).

The complexity of the dynamical processes associated to the multispecies fisheries together with the lack of fisheries production data series increase our difficulty on evaluating the impact of fishing on the natural stocks of the Amazon [73–74]. In multispecies fishery, catch levels tend to increase with the increase of the fishing effort, until a stabilizing point where one cannot increase the production with an increase in effort [75]. It is also known that, contrary to what is observed in fisheries with a single target species which shows a linear drop proportional to the CPUE with an increased effort, the multispecies fisheries CPUE behaves in a non-linear manner [76]. The same occurred in our results: although the annual average of fishing effort (number of fishermen times fishing days) was kept practically stable or was reduced in the period from 1993 to 2003 in Santarém, the CPUE increased probably due to other factors. These factors may be related to technological advancements in the fishing fleet, such as the increase in the average size and engine power of the boats and/or a better ice storage capacity for fish catch preservation [71]. These factors increase the fishing power and are not accounted for in this paper.

Some species of the families Serrasalmidae, Pimelodidae and Scianidae are known to have being caught in the Lower Amazon region in sizes below the average length of the first sexual maturation—L_50_ [76–78], a practice against the current Brazilian law that may compromise the maintenance of these fish stocks in the region. A substantial threat to the sustainability of the fisheries resources in the region is the excessive concentration of fishing effort on just some few target species [76]. In this regard, efforts to diversify the composition of the species caught may reduce the pressure on the species already highly exploited. Meanwhile, despite these considerations, the results of our analyses indicate that the fisheries resources in the Lower Amazon appear to be moderately exploited, that is, below the maximum expected yield [76].

The fishing activity performed in rivers is more complex that the one performed in lakes in the Amazon. The Pimelodidae production, for instance, is practically entirely directed to the industry for processing and is considered the most professional fishing activity of the Lower Amazon region. As a consequence, the Pimelodidae fishing is more influenced by the market than by the environmental factors. The multiple regression models used here are consistent with this reality showing that there is an increase of the Pimelodidae production associated with the market index increase. The opposite happens with the other fishing families that attend the local market: when the market index increases their production decreases.

Although local fishing in lakes near Óbidos is more opportunist and diverse, the local fishing market of Ódibos is very small in comparison to Santarém and Monte Alegre. The fishing production of lake environments in Óbidos e Monte Alegre do not depend on the overall market (industry related) for being typically directed to local consumption. Our results showed that the market index was only important for the Hypophthalmidae and Anostomidae families. The correlations were low with the exception of the fishing of Pimelodidae in river environments. We conclude that the more artisanal the fisheries are, the higher are the errors associated to our statistics to correlate fishing productivity with the environmental variables.

The relative importance of the fish processing industries in Óbidos and Santarém, as well as the fish stocks composition in those sites were previously studied by [49]. The authors used both Principal Components Analysis and a General Linear Model and conclude that the fisheries in those two sites is highly conditioned to the catches of catfish (Pimemolidae and Hypophthalmidae). Moreover, the fisheries of those taxonomic families to feed the industry contribute to the overall fisheries variability especially in the river environment. These results are in agreement with our findings when we add the (economic variable) market index in our analysis.

The fisheries in the Lower Amazon region can be divided into two distinct fishery categories: (1) permanent fisheries in the river environment that occurs along the entire year and (2) cyclic fisheries directed towards the lake environment that is not accessible for the fishing fleet during the dry season [61]. Fishing in floodplain lakes, as opposed to fishing in the river, involves a low degree of specialization and occurs throughout the year [71]. The species caught in the river are extremely vulnerable, with a high risk of overexploitation since fishing is more specialized and fisheries production targets a small number of families and target species. In addition to this, since the main fishing exploration area is the river rather than the lakes, river species are not benefited by fishery agreements held by the coastal communities to protect floodplain lakes and flooded areas [79]. Furthermore, fish species that move between flooded areas and rivers depending on the hydrological cycle phase have a greater plasticity and are less vulnerable to the risk of overexploitation [71]. These species tend to be better protected under community fishery agreements. Thus, new fishing management policies should consider these two categories in order to promote differentiated solutions for the ordinance and sustainability of the fishing activity in the Amazon.

However, it is evident that only fisheries regulation policies are not sufficient to obtain better yields in the activity. Alterations in the Amazonian landscape, especially close to the river banks and floodplains, have impacts on the climatic characteristics of the region affecting the hydrological cycle. In this regard, deforestation causes direct and direct harm to the fish populations due to the reduction in the amount and diversity of food available and due to the alteration of the hydrological cycle. Scientific research demonstrates that an increase in forest biomass leads to an increase in fish biomass, with some species increasing their predominance in the fish communities [80–84]. So, for example, the substitution of forest for grazing land should cause changes in fish eating habits due to the reduction in the supply of fruits, seeds and other forms of organic matter originating in the forest. The impact of deforestation on the stability of the aquatic ecosystem is greater than that of fishing, even on ichthyofauna species that do not depend directly on the forest, such as those in the family Pimelodidae [85]. The extent of degradation and modification of the plain landscape should worsen with the cities growth and the economic development in the Amazon. This can become a considerable threat to the Amazonian environment in the long term, compromising the aquatic and fish ecosystems in the region [86–88].

Fisheries in the Lower Amazon are characterized to cover large areas of lotic environments, such as the Lago Grande of Monte Alegre, the Grande de Curuaí Lake and the Parú Lake (Fig 1). The kinematics of the flow and the dynamics of the flooding pulse play important roles in the life cycle of the ichthyofauna in the Lower Amazon, regulating the availability of food and shelter, influencing the reproduction, food and growth of many fish species [69,81–82, 89]. Constant, increasing modifications of the landscape, change in the river flow due to the construction of barriers and other alterations in the aquatic habitat are pointed out as considerable threats, in the long term, to the ichthyofauna and to the fisheries activity in the Lower Amazon region [41, 81–83]. In addition to the impacts of the landscape and hydrological alterations in the environment, [53] describe that side effects on the fish populations include the intensification of natural death processes caused by the diminution of the body growth and the overall recruitment process.

The diversification of the factors responsible for the climate regulation in the Amazon drove this research to make use of the highest number of possible variables in order to understand the climate dynamics of our study region as well as the effects of those on the aquatic system and fisheries variability. The solar energy availability driving the local energy balance is known as an important factor regulating the climate of the Amazonian region [90]. The energy that arrives at the Earth's surface is delivered back to the atmosphere in the form of sensible and latent heat flux. While the first is responsible for the heating, the second one is related to the evapotranspiration process. Another important variable for all tropical regions of the planet is the precipitation or rainfall. This variable drives the variability of other meteorological variables such as the temperature and the relative humidity, being very heterogeneous in the Amazon [90].

Past studies estimate that about 50% of the water vapor condensed and precipitated by the rainfall in the Amazon is locally recycled through the evapotranspiration process while the other 50% is driven into the region from the Atlantic Ocean [90–92]. The interannual variability of the rainfall and the rivers discharge are related to climatic events such as the ENSO. During the El Niño (positive ENOS phase), the rainfall in the Amazon decreases leading sometimes to several droughts. During these periods the position of the Inter tropical convergence Zone (ITCZ) in the Atlantic Ocean leads to a weakening of the Trade Winds blowing towards the continent [93–96].

As described before, the hydrological cycle in the Lower Amazon is very much determined by the climate, and vice versa [97]. This cycle includes energy exchanges, transport of water vapor, rainfall, drainage, infiltration and diverse water storage mechanisms. All these processes depend on the climate and are determinant to the main characteristics of ecosystems which, in turn, affect the dynamics of the ichthyofauna. Climatic variability and the dynamics of the hydrological cycle in the Lower Amazon, as well as the characteristics of the landscape and the amount of aquatic environments and food available affect the distribution and ecology of the fisheries resources and, thus, the behavior of the fisherman and the fish. However, this behavior is also affected by local culture and the growth of the macro economy. These are dynamic aspects, which present marked temporal variations. [98–99] describe that one must consider the temporal and spatial associations of all the variables in order to better determine the relationships between the environmental variables and the fisheries. In the case of this paper, dividing our study region in the Lower Amazon into three different sites (Óbidos, Santarém and Monte Alegre) tried to respect the considerations above mentioned.

Fluctuations in rainfall in the Amazon are partially associated with ENOS events. During El Niño years there is a tendency to produce drought or accentuated ebb while in La Niña years are associated to intense flooding [37, 75, 96, 100–102]. The duration and intensity of each period in the hydrological cycle are determining factors for the ichthyofauna for they use the floodplains for achieving success of recruitment and consequent species survival [103]. [76, 89, 104] describe that the composition of fish communities in the Amazon floodplain each year is related to the duration and intensity of the hydrological cycle in previous years, suggesting an interannual dependence between ecological and environmental variables. The success or failure of a reproductive strategy (seasonal, balanced or opportunist) and the survival of young individuals in the population of fish in the ebb depends on the adaptation capacity of each species to adapt to the seasonal fluctuations of environmental variables [61, 105].

The climatic variability of the Lower Amazon region has a direct effect on fish catch and consequent productivity as it allows or suppresses the fisherman's access to ideal fishing locations to target the different fish species. The results of all analysis made in this paper indicates that the river-atmosphere-ocean system effects the multispecies fisheries in a different way for each studied region of the Lower Amazon, as well for each fish taxonomic family and for each target species. [106] describe that the temperature acts on the fish growth; the winds, humidity, vegetation coverage and the ENSO events promote or suppresses the availability of food, while the hydrographic catchment, yield in the Amazon River and the surface runoff drive the extent of the flooded area. So, due to the ecological and behavioral complexity of the Amazonian fish resources, the environmental variables can individually or collectively affect their life cycles.

Attested by our environmental data (time series not shown), 1995 was an atypical year during the period of our study. The year of 1995 was characterized by an intense drought in the Amazon basin, leading to a considerable reduction of the aquatic environment area. This must have left the ichthyofauna individuals more vulnerable. In 1995, ENSO was in its positive phase (El Niño) but the event was considered weak progressing to a La Niña in the next year. During that year though, our data (not shown) indicate that there was an atypical increase in sea surface temperature in the Atlantic Ocean, a decrease in the Trade Winds intensity and a consequent reduction in the water vapor transport in the eastern portion of the Amazon Basin [97, 106]. During 1995, a lowering in the annual averaged CPUE occurred in the floodplain lakes.

**Fig 10:**
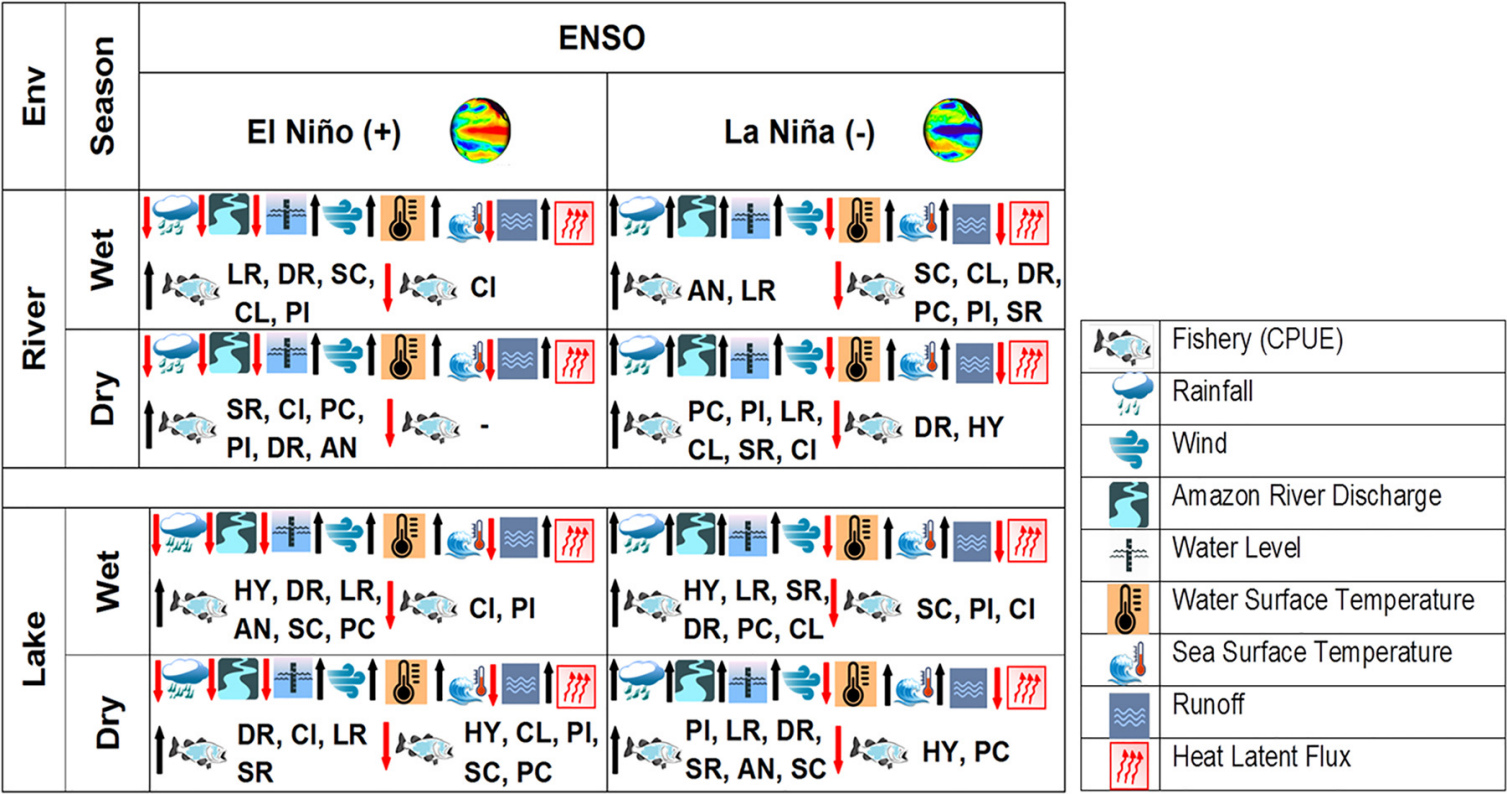
Logical schematic diagram illustrating the dependence relations between the CPUE, ENSO phenomena and environment variables. Logical schematic diagram illustrating the dependence relations between the CPUE of all families, the environmental variables studied here and the persistence of positive or negative ENSO phenomena. (AN) Anostomidae, (CI) Cichlidae, (CL) Clupeidae, (DR) Doradidae, (HY) Hypophthalmidae, (LR) Loricariidae, (PC) Prochilodontidae, (PI) Pimelodidae, (SC) Sciaenidae, (SR) Serrasalmidae.

We assume that during 1995 in the drought event, the accessibility to the floodplain lakes for fishery was more difficult, consequently stimulating the fish activity and an increase in productivity in the river environment. This fact is evidenced in our data by the variability in the CPUE of the taxonomic family Hypophthalmidae. To resume these ideas, we acknowledge that the effects of the climatic (environmental) variability on the Lower Amazon fisheries are important and follow a logic based upon statistical dependence. These effects can be expressed by many logical ways. In Fig 10 we present a logical schematic diagram to illustrate and resume the findings of this paper with respect to the dependence relations between the CPUE of all species, the environmental variables studied here and the persistence of positive or negative ENSO phenomena. These relations are shown in respect to the different seasons (dry or wet) and the fishing environments (river or lakes and floodplains) of our study area. This simple, classic diagram presents the variables and their direct or inverse relations of dependence indicated by the up or down, respectively, direction of arrows.

At the present we know that over the time the amount of water retained in the Amazon Basin has been altered, as well as the greater part of its surface vegetation coverage due to long term geophysical factors [107]. Interannual variability of the flood pulse and other environmental parameters, such as air and surface temperature, have a considerable effect on the diversity of local fauna [108] and, as a result, on the fisheries activity and production [89]. Higher temperatures reduce the solubility of oxygen in water, which may increase the demand for oxygen and food consumption of fish due to their increased metabolic rate. The increase of the temperature in the surface layer of the water may also promote the development and survival of parasites and bacteria. These facts combined together can reduce the survival, growth and reproductive success of fish populations [109].

We also know that the ichthyofauna in the floodplain lakes of the Lower Amazon have a complex behavioral dynamic. During the wet period, following reproduction, larvae and/or young fish of many fish species are carried out by the river current into the flooded areas [19, 61, 66, 110–111], a free aquatic environment where they should search for food and shelter against various types of predator. Even during the dry season, fish adults try to move into the rivers since the aquatic environments are concentrated in the main channels [61]. This particular dynamic is echoed in fish productivity and in the behavior of the fishermen. The predicable effects of drought in the Amazon in global climate change scenarios include an estimated loss of 7 to 12% of fish species by 2070 in the Amazon River Basin [111].

## Conclusion

Environmental variables such as the air, water and soil temperatures, atmospheric humidity, the water level, the local winds as well as climatic variables measured indexes such as the MEI, NOA and GITA act at distinct temporal scales promoting an clear effect on the multispecies fisheries of the Lower Amazon region;

The relationship between the monthly average CPUE of different taxonomic family of target fish presents different behavior depending on the environmental or climatic variable considered;

The fishery productivity in the study area presents different patterns in respect to the environment and climate depending if it is originated from the river or from the floodplain lakes. A clear variability occurs both along the River Amazon extend in the Lower Amazon (longitudinal gradient) and laterally across the river (latitudinal gradient) for each kind of fishery studied here. This depends on the ecological characteristics and life strategies of each target taxonomic family considered in the fishery;

The intensity of fish exploration in the Lower Amazon region is apparently still moderate;

The preservation of the landscape and the equilibrium of the energy and hydric balances should be considered for future fish management policies;

New programs aiming the long term collection and processing of fishery statistics data in the Amazon River Basin should be put forward and incentivized. In a Global Change scenario with perceived changes in the vegetation cover of the Amazon region we believe that the strengthening of our knowledge about the relationship of the environmental and climatic variables with the fisheries is crucial for any future environmental modeling of the region;

Future research should also drive attention towards the identification of new variables to be included into the multivariate models. Possible new environmental variables should include the positioning and frequency of the instability lines of clouds in the atmosphere responsible for the region's rainfall patterns, the Inter Tropical Convergence Zone and the Bolivian Height positioning and persistence, the fluvial breeze, water quality variables (for example the phytoplanckton, nutrients, pollutants and dissolved oxigen concentrations). Ideally, new fisheries data should be produced in a better georefering interface such as a Geographical Information System.

## Supporting Information

S1 Dataset CPUE river dataset. Dataset of CPUE in the river for fishing grounds in Óbidos, Santarém and Monte Alegre and taxonomic families of fish. (AN) Anostomidae, (SR) Serrasalmidae, (CI) Cichlidae, (HY) Hypophthalmidae, (LR) Loricariidae, (PI) Pimelodidae, (PC) Prochilodontidae, (SC) Sciaenidae, (DR) Doradidae, (CL) Clupeidae.
(XLSX)

S2 Dataset CPUE lake dataset. Dataset of CPUE in the floodplain lakes for fishing grounds in Óbidos, Santarém and Monte Alegre and taxonomic families of fish. (AN) Anostomidae, (SR) Serrasalmidae, (CI) Cichlidae, (HY) Hypophthalmidae, (LR) Loricariidae, (PI) Pimelodidae, (PC) Prochilodontidae, (SC) Sciaenidae, (DR) Doradidae, (CL) Clupeidae.
(XLSX)

S3 Dataset Market index for each fishing ground. Market index considering river and floodplain lakes for Óbidos, Santarém and Monte Alegre fishing grounds.
(XLSX)

S4 Dataset CPUE lake dataset. Time series of environmental variables and climate indeces. (ARD) Amazon River Discharge, (WL) Water Level, (RF) Rainfall, (u) Zonal Wind Component, (v) Meridional Wind Component, (TEMP) Surface Temperature, (SPFH) Specific Humidity, (RUNOF) Runoff, (LHF) Latent Heat Flux, (SSTA) Sea Surface Temperature, (MEI) Multivariate ENSO Index, (GITA) Atlantic Inter-Hemispheric Sea Surface Temperature Gradient, (NAO) North Atlantic Oscillation.
(XLSX)
